# Motion Artifact Reduction for Wrist-Worn Photoplethysmograph Sensors Based on Different Wavelengths

**DOI:** 10.3390/s19030673

**Published:** 2019-02-07

**Authors:** Yifan Zhang, Shuang Song, Rik Vullings, Dwaipayan Biswas, Neide Simões-Capela, Nick van Helleputte, Chris van Hoof, Willemijn Groenendaal

**Affiliations:** 1 Holst Centre/imec, 5656AE Eindhoven, The Netherlands; z.yifan1@gmail.com; 2 Department of Electrical Engineering, Eindhoven University of Technology, 5600MB Eindhoven, The Netherlands; r.vullings@tue.nl; 3 imec vzw, 3001 Leuven, Belgium; dwaipayan.biswas@imec.be (D.B.); neide.simoescapela@kuleuven.be (N.S.-C.); nick.vanhelleputte@imec.be (N.v.H.); chris.vanhoof@imec.be (C.v.H.); 4 Department of Electrical Engineering, KU Leuven, 3001 Leuven, Belgium

**Keywords:** photoplethysmography, motion artifacts, heart rate, continuous wavelet transforms

## Abstract

Long-term heart rate (HR) monitoring by wrist-worn photoplethysmograph (PPG) sensors enables the assessment of health conditions during daily life with high user comfort. However, PPG signals are vulnerable to motion artifacts (MAs), which significantly affect the accuracy of estimated physiological parameters such as HR. This paper proposes a novel modular algorithm framework for MA removal based on different wavelengths for wrist-worn PPG sensors. The framework uses a green PPG signal for HR monitoring and an infrared PPG signal as the motion reference. The proposed framework includes four main steps: motion detection, motion removal using continuous wavelet transform, approximate HR estimation and signal reconstruction. The proposed algorithm is evaluated against an electrocardiogram (ECG) in terms of HR error for a dataset of 6 healthy subjects performing 21 types of motion. The proposed MA removal method reduced the average error in HR estimation from 4.3, 3.0 and 3.8 bpm to 0.6, 1.0 and 2.1 bpm in periodic, random, and continuous non-periodic motion situations, respectively.

## 1. Introduction

Photoplethysmography (PPG) is a widely used non-invasive optical sensing technology to monitor the cardiovascular and respiratory systems. PPG can measure changes in tissue and blood volume by emitting light on tissues and detecting the variations in optical absorption and scattering. The applications of PPG include monitoring of heart rate (HR), hemoglobin oxygen saturation (SpO2), and potentially detection of epileptic seizures and atrial fibrillation [1,2,3]. Meanwhile, the use of wearable devices such as wrist-bands, smartwatches and health patches for vital sign monitoring is growing. In particular, wrist-worn wearable devices with integrated PPG sensors are becoming increasingly popular for ambulant HR monitoring. Compared with electrocardiography (ECG) or fingertip PPG, wrist-worn devices with reflective detecting mode have a small form factor and high user comfort since it does not require wet electrodes or a finger clip.

During ambulatory monitoring various types of motions, such as walking, stretching and finger tapping, are present that can distort the measured PPG signal. Motion artifacts (MAs), which can be periodic or non-periodic, can have much larger amplitude than the pulsatile component in the PPG. In addition, the MAs can be in the same frequency range as the HR signal [4]. Compared to PPG from the finger, PPG signals measured on the wrist can suffer from more intensive and complicated MA due to the flexibility of wrist. Therefore, motion artifact reduction (MAR) for these devices is challenging. In Section 2.2 we review the different strategies for MAR. 

Most existing solutions for MAR use a motion reference signal, often from an accelerometer [2,3]. More recently the use of a gyroscope as a motion reference has been explored [5,6,7]. Though promising results have been obtained for MAR for different types of motion, MA resulting from micromotions are still challenging. MA arising from fine-grain movements/gestures are not accurately captured in the accelerometer recordings. For example, while tapping one of the fingers, the wrist can stay still, which makes accelerometers integrated in a wrist band ineffective as a motion reference. These fine-grain movements have a large effect on wrist PPG signal quality. Therefore, a photoelectric motion reference that is affected similarly by the MA can be a better motion reference. Moreover, a multichannel PPG sensing strategy could also account for the variability in signal quality resulting from how the device is worn across individuals [8,9].

Another comparison between accelerometer and the IR PPG channel is with respect to system design. Although, an accelerometer consumes around 10% to 20% of the power consumed by the IR PPG channel, adding an accelerometer complicates the systems design and increases the formfactor. The IR PPG channel is typically available in most PPG sensors and readout systems. 

A modular algorithm framework for MAR based on PPG obtained by different wavelengths is proposed in this paper. This approach is verified using data of six heathy subjects performing 21 types of motion. The results show clear improvement on the average error in HR estimation during periodic, random and continuous non-periodic motion situations. The paper is organized as follows. In Section 2, the origin of micromotion artifacts in PPG signals is explained and existing MAR algorithms are reviewed. In Section 3, the used dataset is described and the properties of the signals recorded by wrist PPG sensors are analyzed. With the obtained knowledge on the MA components in PPG recorded by green and infrared (IR) light, several signal features of the MA and PPG are extracted, which will be exploited in the proposed algorithm framework. In Section 4, a modular algorithm framework for MAR is proposed. After that, in Section 5, the evaluation results are presented in terms of HR error rate. Finally, conclusion and future recommendations are provided in Section 6.

## 2. Micromotion Artifacts

The focus of this study is on MAR for ambulatory HR monitoring using wrist-based PPG. Specifically, a group of motions called micromotions, such as finger tapping and fist opening and closing, are investigated. In this section, the origin of the MA is explained together with the justification for using the IR PPG signal as the motion reference. In addition, relevant work on MAR algorithms is reviewed. 

### 2.1. Photoelectric Motion Reference

Reflective detection mode is usually used in wrist-worn PPG sensors. The principle of reflective detection is described by the Beer-Lambert law as shown in Equation (1), where *I_i_* and *I_o_* are the input and the reflected output light intensity respectively, *c* represents the concentrations of different tissues, *d* represents the reflective light path length and *ε* represents the reflection/absorption coefficient of the different tissues. The incident ray passes through different layers of tissues and is reflected by them. The *ε* and *d* also depends on the wavelength of light, while the *c* and *d* can be time varying in case of motion [10]:

(1) $$I_{o}=I_{i}\cdot exp\left(-\overset{n}{\underset{j=1}{\sum }}\epsilon_{j}\cdot c_{j}(t)\cdot d_{j}\left(t\right)\right)$$

**Figure 1:**
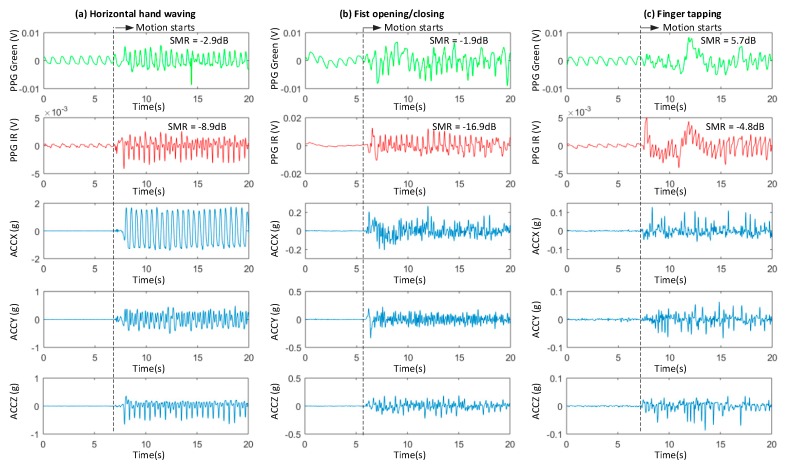
Recorded Green/IRPPG and 3-axis acceleration data (X, Y and Z) during 3 types of periodic motion: (**a**) Horizontal hand waving; (**b**) Fist opening/closing; (**c**) Finger tapping (index finger). Note that the scales of the y-axes are different, due to different magnitudes of the signals. The signal to motion ratio (SMR) is embedded.

According to Equation (1), there are two main sources of the MA in PPG signal. First, the displacement of the sensor relative to the skin leads to a change in the incident angle and light path, as expressed as *d_j_* (*t*). Second, the internal deformation and structural change of certain tissue, as expressed as *c_j_* (*t*), result in an artifact in the signal. For wrist-based devices, an accelerometer can record *d_j_* (*t*), however it cannot record *c_j_* (*t*), because *c_j_* (*t*) does not involve motion of the wrist. Figure 1 shows green PPG, IR PPG and 3-axis acceleration data (X, Y and Z) during three types of 2 Hz periodic motion. The MA caused by hand waving motion can be detected very well by the accelerometer, showing a clear 2 Hz periodic component in ACCX data (Figure 1a). The MAs caused by fist opening/closing and finger tapping are more difficult to detect by the accelerometer, because the wrist (wearing the device) of the test subject can stay still (Figure 1b,c). Although less clear, some periodic motion signal can still be seen in ACCZ for fist opening and closing (Figure 1b). No stable periodic signal can be found in any of the three axes of the accelerometer during finger tapping (Figure 1c). On the other hand, in all three cases, the IR PPG signal contains clear periodic motion component at 2 Hz, indicating that it could be used as motion reference during micromotions. 

In order to improve the effectiveness of motion recording during micromotions, a photoelectric motion reference originating from the same source of the MA is chosen instead of an accelerometer. It is well known that the depth of penetration of light into human skin increases with reducing wavelength [11]. The green light reaches mainly the capillary layer and thus results in a higher AC/DC ratio of the obtained PPG signal than the signal obtained by red and IR light, which can penetrate deeper. According to Equation (1), the motion artifacts in the IR PPG signal have much larger amplitude due to a larger change in light path d, compared to the MAs in the green PPG signal [11,12]. This is also shown in Figure 1, the signal to motion ratio (SMR) is at least 10 dB higher in green PPG than in the IR PPG. 

Therefore, we propose to use a PPG signal recorded by green light as the main channel for HR detection and a PPG signal channel recorded by IR light as the motion reference. Typically, both are available in PPG sensors [13]. In Section 3, the correlation between MA in the main PPG signal (green PPG) and in the motion reference (IR PPG) during different motions is investigated.

### 2.2. Motion Artifacts Reduction Algorithms

In order to extract physiological parameters, e.g., HR, from PPG signals corrupted by MA, recovering artifact-reduced PPG signals has been extensively researched [2,3]. Prior work on MA removal can be categorized as: (1) methods using single channel PPG signal; (2) using multi-channel PPG signals and (3) using PPG signal together with a motion reference.

Algorithms based on single channel PPG signals have generally used time-frequency decomposition methods to separate the MA component from corrupted signal. For example, Zhang et al. [2] use singular spectrum analysis (SSA) to decompose a raw PPG signal into several components and then use sparse signal reconstruction (SSR) to find the heart rate component. Besides SSA, empirical mode decomposition (EMD), wavelet decomposition, single measurement vector (SMV) and multiple measurement vector (MMV) methods are used [14]. In general, these algorithms work relatively well with non-periodic MA. However, when the motion is periodic and intensive, the accuracy usually decreases since in that case it is very difficult to distinguish the PPG signal and MA.

Algorithm approaches for MAR in literature using multi-channel PPG signals, like independent component analysis (ICA), separate the HR signal and the MA in the time-domain, based on the fundamental assumption of statistical independence between MA and the clean PPG signal. These signals are not always statistically independent since motion can affect arterial flow, yielding a pulsatile PPG component. Hence, these algorithms are less effective in such situations. A multi-channel PPG MAR method was considered in [15,16,17,18]. Reference [15] considered IR and red channels in a finger oximeter, [16,17] considered two green channels in conjunction with WPPG, [18] focused on two infrared channels with the sensor placed on the ear, whereas [19] used as many as nine channels for sensing PPG.

Attempts have been made to remove MAs by using an additional motion reference signal to reduce the MA component from the PPG signal. Most often an accelerometer has been used as a motion reference [2,3,4]. However, recently the use of a gyroscope as motion reference was explored. These studies indicated that the gyroscope allows for the separation of acceleration due to the movement of the sensor or due to gravity and can be used as a motion reference for MAR [5,6,7]. Algorithms in this category include time domain and frequency domain motion reduction. In time domain, adaptive filtering methods such as Least Mean Square (LMS) and Recursive Least Squares (RLS) are commonly used. In frequency domain, Spectrum subtraction (SS) is a widely adopted approach. These methods depend strongly on the high correlation between the MA in the PPG signal and the motion reference signal [20,21]. However, as discussed in Section 2.1, certain types of micro/fine-grain motions cannot be recorded effectively by accelerometers, which motivates the choice of using a photoelectric motion reference. To the best of our knowledge, the proposed approach is the first to use a different wavelength channel as the motion reference for wrist-worn PPG sensor systems. The signal properties of this motion reference and the developed algorithm framework are discussed in Section 3 and Section 4, respectively.

## 3. Dataset and Signal Property

The dataset and measurement setup used in this work are described in Section 3.1 and the characteristics of the MA and the HR components in the green and the IR PPG signal are analyzed in Section 3.2. The correlation between MAs in the two PPG signals, the difference in signal to MA ratio and the distribution of the difference in HR between adjacent time segments are presented and discussed. These properties will be exploited in the proposed algorithm framework in Section 4. 

### 3.1. Dataset and Measurement Setup

**Figure 2:**
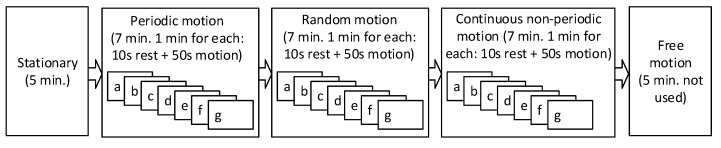
The structure of one dataset including stationary, periodic, random and continues non-periodic motion segment.

The dataset includes data from six healthy measurement subjects with age from 25 to 35. The data are collected at Holst Center (Eindhoven, the Netherlands) on a voluntary basis. The declaration of Helsinki has been followed during this work and all measurement subjects gave informed consent. Each subject performed seven types of motion in periodic, continuous non-periodic and random manner, resulting in 21 motion sub-types. The structure of one dataset is shown in Figure 2. The focus of this dataset is on micromotions, where the trunk of the human body stays still. In addition, there is a 5 min stationary part before the motion starts. The included motions are:

(a) Index finger tapping
(b) Hand waving (horizontal)
(c) Hand shaking (vertical)
(d) Running arm swing
(e) Fist opening and closing
(f) Radial/ulnar deviation
(g) Wrist extension/flexion

The dataset includes one channel of ECG (lead II configuration) recorded from the chest. The ECG together with one channel of finger PPG are recorded by a g.USBamp (g.tec medical engineering GmbH, Schiedlberg, Austria) signal acquisition system. The ECG is used as the reference for HR and the finger PPG is used only for signal alignment In addition, two channels of PPG signals using green (wavelength: 660 nm) and IR (wavelength 940 nm) LEDs were recorded with a wristband. The two channels of wrist-worn PPG signals are recorded by the AFE4403 evaluation board from TI@ (Texas Instruments, Dallas, TX, USA) together with a SFH7050 integrated PPG sensor (OSRAM Licht AG, Munich, Germany). At the start of the data collection, an ambient pulse signal is applied that is picked up by both finger and wrist PPG sensors. This ambient pulse is used for synchronization between the two systems, i.e., between the ECG reference and the wrist PPG signals.

### 3.2. Signal Property

In order to analyze the MAs in the green and infrared PPG, the MAs need to be extracted from the original recorded PPG signals. In our experiments, the ECG signal is collected to provide a reference for the HR (HR_Ref_), and the instant HR is estimated by ECG R-peak detection. The HR_Ref_ is used to set the frequency band for a bandpass filter that is then applied to the PPG signal to recover the clean PPG signal. The clean PPG signal is composed of the fundamental HR component together with its second and third harmonics. The rest of PPG signal is considered as MA. The following analysis is based on data from all six subjects. 

#### 3.2.1. Correlation between MA in Green and IR PPG Signals

**Figure 3:**
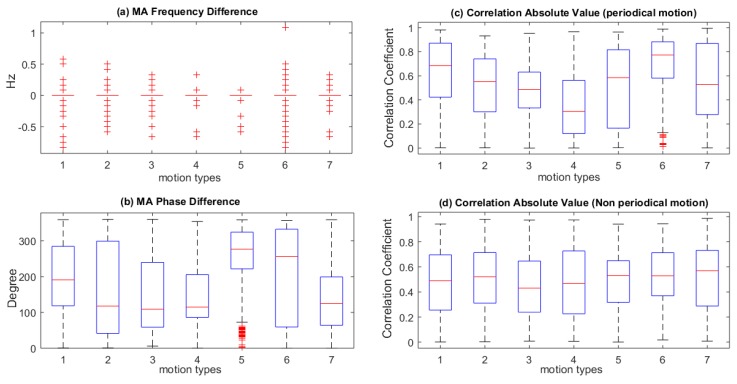
Correlation between MA in green and IR PPG: (**a**) Boxplots of the frequency difference between the frequency of the MA in the green and the IR channels for the seven types of periodic motion. Due to similarity in these frequency components the median, interquartile range and whiskers overlap. The outliers are visible.; (**b**) Phase difference between the MA in the green and the IR channel during the seven types of periodic motion; (**c**) Correlation coefficient between the motion in the green and the IR channel during the seven types of periodic motion; (**d**) Correlation coefficient between the motion in the green and the IR channel for the seven types of continuous non-periodic motion.

The correlation between the MA in the green and in the IR PPG channel is critical for effective motion artifact reduction. Firstly, seven types of periodic motions are analyzed in terms of frequency and phase difference between the extracted MA in the green channel and the IR channel (Figure 3a,b). The MA fundamental frequency and phase differences are calculated in 47 consecutive 4 s windows during one minute (50 s motion with 3 s overlap, excluding 10s rest time at the start). The results are illustrated with boxplots showing the median, the third quartile, the whiskers (+/− 2.7σ) and outliers by using default settings in Matlab@ (Mathworks Inc., Natick, MA, USA). Each motion type in Figure 3 contains 282 (47 windows by 6 subjects) segments. The fundamental frequencies of the MA in the green and the IR are very similar as shown by the overlap of the median, the interquartile range and whiskers. The outliers are visible (Figure 3a). This similarity in fundamental frequency of the MA in the green and the IR channel will be exploited further in Section 4. On the other hand, Figure 3b shows a large variance (>45 degree) in all periodic motion types, which indicates a low time domain correlation.

Secondly, the time-domain correlation coefficients between the two motion signals (green and IR) for seven types of periodic and continuous non-periodic motion are analyzed (Figure 3c,d). The random motion situation is not included in this analysis, because the correlation coefficient is dependent on the portion of motion segment, which varies in different motion types and measurement subjects. The correlation coefficients were calculated in the aforementioned 47 segments of 4s time windows. The analysis shows that the time domain correlation between the MA in the green and IR PPG fluctuates significantly and that the correlation is not sufficient for MAR in the time domain, e.g., by adaptive filtering. 

#### 3.2.2. Clean PPG Signal to MA Ratio

**Figure 4:**
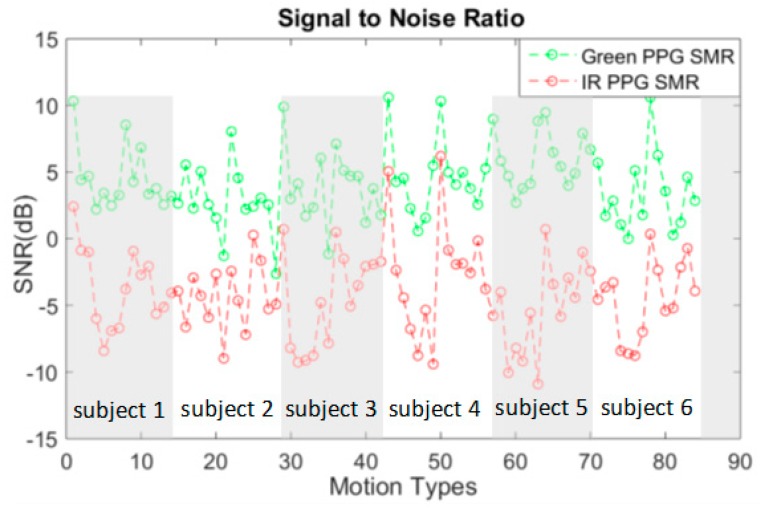
The SMR of green and IR PPG in different motion types among six subjects.

The ratio between the clean PPG signal to the MA components in green and IR channel are calculated. Each of the seven motion types in periodic and continuous non-periodic from all six subjects are used in this calculation, resulting in 84 data points (14 submotion types by six subjects) in Figure 4. The random motion situation is not included for the same reason discussed in Section 3.2.1. It can be observed that in most situations the signal to MA ratio (SMR) of green PPG is 10 dB higher than the one of IR PPG. This indicates that the PPG recorded by IR is indeed more sensitive to the MA, and thus can be used as a motion reference.

#### 3.2.3. Heart Rate Changing Variation Distribution

**Figure 5:**
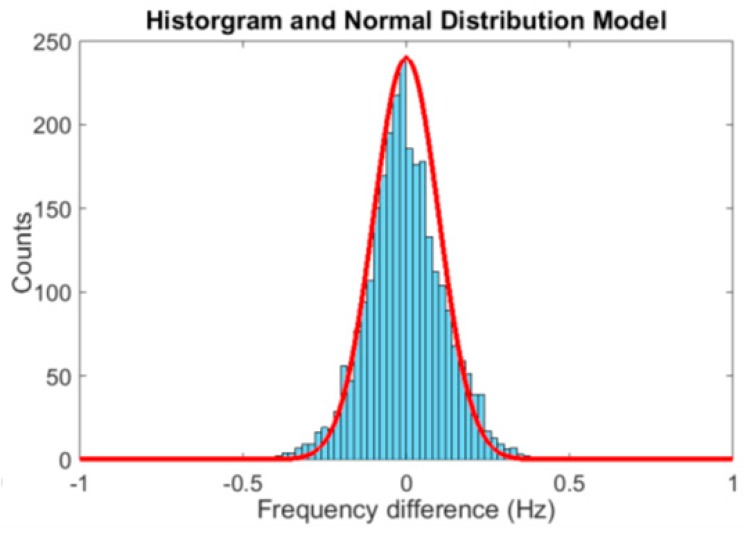
The histogram of average HR frequency variance calculated in adjacent 4s windows.

For healthy subjects, the difference in the average HR between adjacent segments of less than 10 s is usually very limited [2,22]. The difference in average HR between two adjacent segments of 4 s is analyzed within our dataset for a total signal length of over 180 min (2700 segments of 4s) recorded from six subjects. The difference in average HR between adjacent segments can be described with a normal distribution with a standard deviation of 0.1 Hz, expressed as X~N (0; 0.1) (histogram in Figure 5). This feature will be exploited in HR frequency tracking in Section 4. Based on these features, a modular algorithm framework is proposed in Section 4 for motion reduction.

## 4. Proposed Algorithm Framework

**Figure 6:**
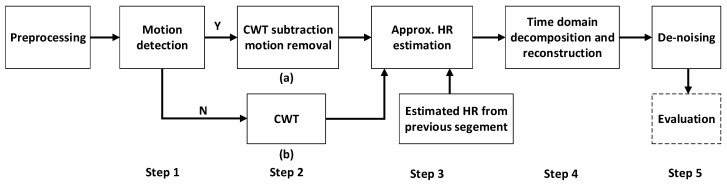
The proposed algorithm framework for MAR based on two wavelengths including five steps.

The proposed algorithm framework for MAR based on two wavelengths is shown in Figure 6 where the blocks indicate the different processing steps. This algorithm framework takes the IR PPG channel as the motion reference together with the green PPG channel for HR extraction. There are 5 steps in this framework, which will be discussed in Section 4.1, Section 4.2, Section 4.3, Section 4.4 and Section 4.5. Step 1 is preprocessing by bandpass filtering together with motion detection based on signal properties including peak to average ratio and the AC/DC of the PPG signals. In step 2a, the MAR is performed by normalized spectrum subtraction after continuous wavelet transform (CWT). When no motion is detected in step 1, the CWT of green PPG signal will be the output spectrum of this step (2b). In step 3, the HR is estimated taking into consideration both the HR from the previous segment and the spectrum from the current segment obtained in step 2. In step 4, a frequency mask based on the HR estimate from step 3 is obtained for signal reconstruction. This frequency mask is then compared with each component after singular spectrum analysis (SSA) decomposition to decide which components to use for signal reconstruction. Finally, in step 5, de-noising is applied in the time domain. 

### 4.1. Preprocessing and Motion Detection

In order to cancel the effect of light intensity, the signal is divided by its DC component that is proportional to the light intensity. The DC component is calculated by means of the recorded PPG signals of green and IR light during the stationary situation [23]. Next, both the green and IR signals are filtered with a second order IIR bandpass filter (0.4–4 Hz) to remove the noise and MA outside the normal range of human HR [4].

Before entering step 2, it is needed to detect the presence of motion to avoid removing the actual HR component in stationary situations. Motion detection is based on two parameters: (1) The peak to noise ratio of the green PPG signal in the frequency domain; (2) The ratio between the AC/DC of the green PPG signal and the AC/DC of the IR PPG signal. 

The peak to noise ratio of the green PPG signal is much higher during stationary situations and periodic motion situations than during the other motion situations. In the first two situations, the signal has a clear periodic component (either HR or MA component) while in the other two motion situations, the MA component can spread over frequency spectra, leading to an increase of noise. The ratio (of the AC/DC) between green PPG and IR PPG is an indicator to separate the periodic motion situation from stationary situations. Typically, during stationary situations, the AC/DC of the green PPG channel is usually more than 10 times higher than the AC/DC of the IR PPG channel [23]. On the contrary, during motion, the MA component in the IR PPG signal is much larger than the MA in the green channel as discussed in Section 3.2.2.

In general, both a higher peak to noise ratio and a higher power ratio between green and IR signals indicate a stationary situation, while lower values of both parameters point to situations with motion. A support vector machine was trained using these two features to classify the signal into parts with and without motion.

### 4.2. CWT-Based Motion Artifact Removal

As discussed in Section 3.2.1, the MAs in the IR PPG signal have good frequency similarity with the MAs in the green PPG signal during periodic motion. Moreover, the ratio between MAs and clean PPG signal in IR PPG is much higher than in green PPG. Therefore, the HR component of the green PPG signal can be enhanced, by removing the MA component from the green PPG signal by subtracting a scaled IR signal from the green signal in the frequency domain. The algorithm needs to deal with different types of motions in daily life, which are not always periodic. Therefore a signal analysis method in the time-frequency domain is needed. We propose to use continuous wavelet transform (CWT), which has higher accuracy in both time and frequency domain compared to short time Fourier transform (STFT). 

In the frequency domain, the spectrum of the corrupted PPG signal, *P_m_*(*f*), can be expressed with Equation (2), where *P_c_*(*f*) is the PPG HR component and *MA*(*f*) is the motion component. The frequency domain MA removal can be expressed using Equation (3), where *P_cG_*(*f*) and *P_cIR_*(*f*) are the spectra of respectively the green and IR HR components and *MA_G_*(*f*) and *MA_IR_*(*f*) are the spectra of the MA component of respectively the green and IR signals. The green and IR spectra are normalized before subtraction, resulting in an adaptive filter. Scaling factor W is set by the ratio between the largest components in the spectra of the IR and the green PPG signals:

(2) $$P_{m}\left(f\right)=P_{c}\left(f\right)+MA\left(f\right)$$

(3) $$P\left(f\right)=P_{cG}\left(f\right)+MA_{G}\left(f\right)-W\cdot (P_{cIR}\left(f\right)+MA_{IR}\left(f\right)$$

**Figure 7:**
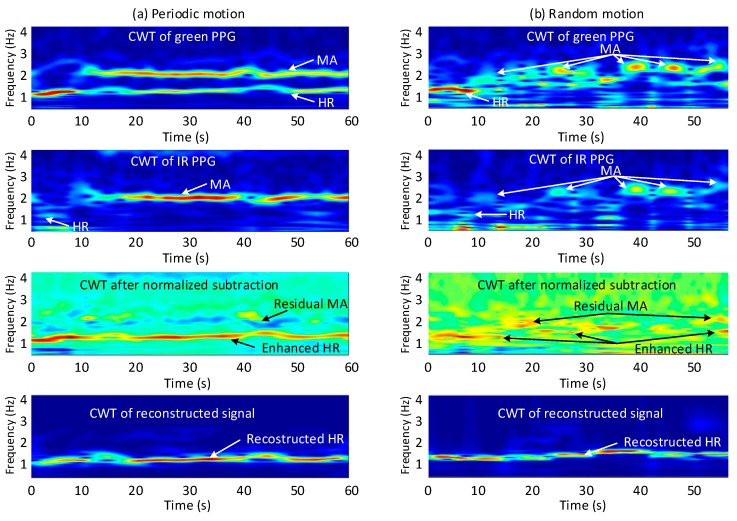
Motion reduction based on CWT: (**a**) Periodic motion type g; (**b**) Random motion type g. The first row shows the CWT of the green PPG signal during these two types of motion. The second row depicts the CWT of the IR PPG signal. The third row gives the CWT after normalized subtraction (Equation (3)). The last row shows the CWT of the reconstructed signal.

Examples of periodic and random non-periodic motion are shown in Figure 7a,b. From top to bottom, the CWT of the green PPG signal, the CWT of the IR PPG signal, the CWT of the green PPG signal after subtraction and the CWT of the reconstructed green PPG signal are illustrated. Comparing the CWT of green and IR PPG in Figure 7a, the ratio between the HR component and the MA for the green channel is indeed much larger than the ratio for the IR channel as discussed in Section 3.2.2. During both periodic motion and random motion, the MA components are reduced, and the HR components are enhanced after CWT spectrum subtraction (third row in Figure 7). After time domain signal reconstruction (will be discussed in Section 4.4), the HR component can be clearly seen in the CWT spectrum the whole time (final row in Figure 7). What is more, during the stationary situation (the first 10 s of the signal segment), the aforementioned motion detection classifier prevents the subtraction, and thus the HR components remains intact in the reconstructed signal.

### 4.3. Approximate HR Estimation

In this step, the approximate HR of the current segment is estimated based on both the result of step 3 (CWT-based MA removal) on the current segment and the HR frequency estimated in the previous segment. The HR estimation from the previous segment is obtained for the previous window after step 3. For the first segment of the data, the information from the previous segment cannot be used. Using information from both the current and the previous segment enables tracking of the HR frequency. 

The output of step 2 is a frequency spectrum of a 4 s signal segment, containing in principle only the HR related components of PPG. The amplitude information is used to calculate a probability distribution of HR. The frequency spectrum contains 256 components (from 0.5 Hz to 4 Hz), the sum of which is normalized to 1 by scaling each component with the same factor. Secondly, the HR frequency estimated in the previous segment (*f*_HRpre_) is used to predict the HR in the current segment. The prediction of the HR is based on a normal distribution around the HR estimated for the previous segment, *X*~*N* (HR_estimated_; 0.1). This normal distribution is based on the results of the analysis in Section 3.2.3. and visualized in Figure 5. The difference being, in Figure 5 the distribution is centered around 0, while here the normal distribution with a standard deviation of 0.1 is centered around the HR estimate of the previous segment. 

For these two distribution functions the confidence levels are calculated using two parameters: the approximate entropy (AE) of the signal and the peak to noise ratio calculated in the frequency domain. The AE quantifies the amount of regularity and the random fluctuations in the time domain signals. When the signal contains only clean PPG signal with high regularity, a low entropy can be obtained. On the other hand, signals with more non-periodic MA components will show a higher entropy. Therefore, signal segments with a low entropy and a high peak signal to noise (discussed in Section 4.1) are considered to have higher confidence level.

**Figure 8:**
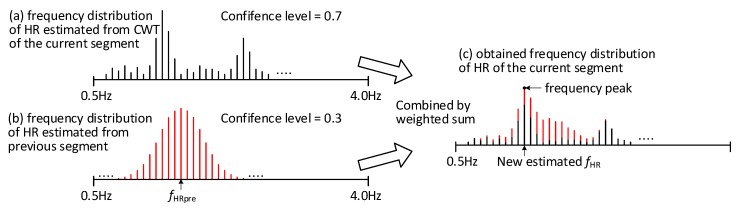
Approximate HR estimation based on weighted sum: (**a**) the frequency distribution estimated from CWT of the current segment, (**b**) the frequency distribution estimated from previous segment (**c**) the obtained frequency distribution of the current segment.

Finally, the sum of these two confidence levels is normalized to 1 and the scaled level is used to combine the information from the previous and the current segment. Thus, a final estimated HR frequency distribution function is calculated as the weighted sum of the two distribution functions as shown in Figure 8a,b. The estimated HR is obtained by finding the frequency peak in this distribution function as shown in Figure 8c.

### 4.4. Signal Reconstruction

HR usually shows beat-to-beat variations, which is relevant information when analyzing heart activities. The estimated HR in Section 4.3 is an average HR within a 4 s segment. Therefore, signal reconstruction in the time domain is needed to provide the peak positions that can be used to obtain beat to beat HR. There are three steps in the proposed signal reconstruction. 

**Figure 9:**
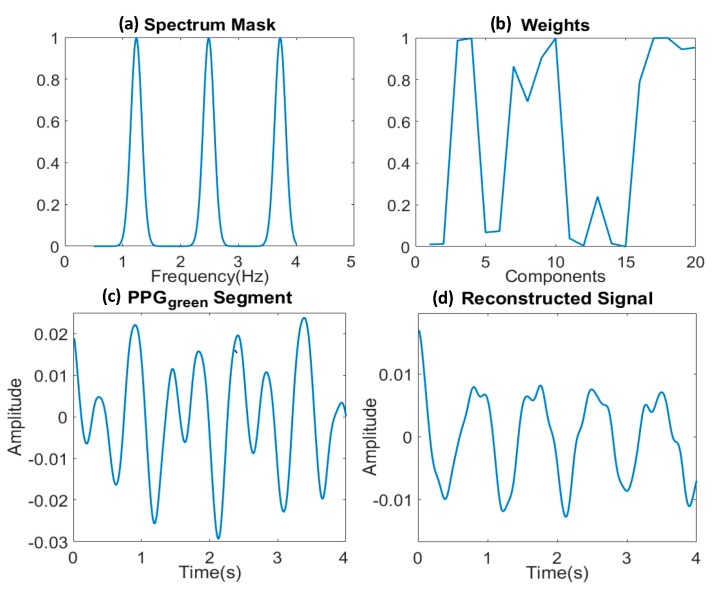
Signal reconstruction step: (**a**) The frequency mask used for signal reconstruction; (**b**) The rescaled weights after SSA decomposition; (**c**) One example of the corrupted PPG signal; (**d**) The corresponding reconstructed PPG signal.

Firstly, the time domain signal of one segment is decomposed into 20 components by singular spectrum analysis (SSA), each component with its own weight. These components contain either HR information or noise. Secondly, the correlation of each component with a frequency mask based on the estimated approximate HR is calculated. This mask, shown in Figure 9a, consists of three normal distributions around the fundamental, the second and the third harmonics of the HR frequency: {*f*_HR_ − σ, *f*_HR_ + σ}, {2*f*_HR_ − σ, 2*f*_HR_ + σ}, {3*f*_HR_ − σ, 3*f*_HR_ + σ}, where σ is 0.1 Hz, as determined in Section 3.2.3. These correlations show the probability of every component from SSA to be part of the clean PPG signal. Then the weight of each component obtained from SSA is rescaled by the correlation of this component with the spectrum mask as shown in Figure 9b. Finally, all 20 rescaled components are summed. The signal reconstruction is also performed in every 4 s window. Figure 9c provides an example of the original corrupted PPG signal, and the corresponding reconstructed signal is shown in Figure 9d, where the HR component can be clearly observed.

### 4.5. De-Noising

**Figure 10:**
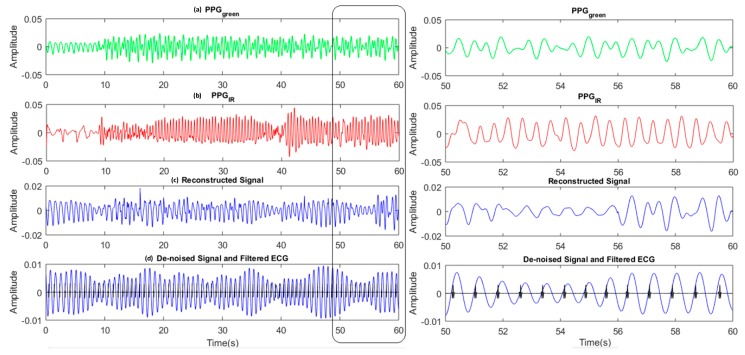
Left: Time domain signals from top: (**a**) Corrupted Green PPG signal and; (**b**) Corrupted IR PPG signal; (**c**) PPG signal after motion removal and reconstruction; (**d**) PPG signal (blue curve) after de-noising together with ECG (black curve). Right: Similar signals as on the right but an enlargement of the signal between 50 and 60 s.

The de-noising step is performed for a longer segment (8 s with 4 s overlapped) to remove dis-continuities caused by the operations of the previous steps in 4s non-overlapping windows. It is basically an SSA operation to remove the noise from the signal. This step also helps to apply the information from the reconstructed signal to the nearby segment. An example is given in Figure 10. The signal reconstruction (using step 4 of the framework) for the period between 50 and 60 s is not ideal (Figure 10c). Using the de-noise step results in a clean PPG signal (Figure 10d) with the help of the SSA, applying information from previous segments. 

**Figure 11:**
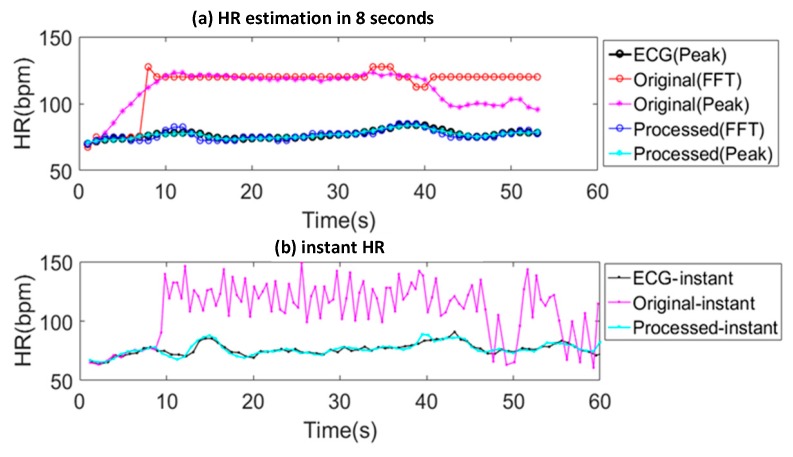
(**a**) The average HRs obtained by ECG, original signal with bandpass filtering and the processed signal using the framework; (**b**) The instant HRs obtained by ECG, original signal with bandpass filtering and the processed signal using the framework.

The effectiveness of this algorithm framework (during motion type g: periodic wrist extension) in terms of average HR in 4 s windows and instant HR is shown in Figure 11. Figure 11a shows the average HRs obtained by ECG as the golden standard, the original signal after step A (bandpass filtering) and the processed signal with the proposed framework. The HRs from the PPG signals are obtained by both frequency domain and time domain methods, namely FFT and peak detection in 4s window. The time domain method uses convolution of the PPG signal with the derivative of a gaussian after which the valleys in the convolved signal are detected. The valleys detected in the green PPG signal are used for HR calculation. The beat to beat HR detection for the ECG data is performed based on peak detection after Mexican hat wavelet transform. 

Both the results obtained by FFT and peak detection on the PPG signal are far from the HR obtained by ECG while the average HRs obtained from the proposed framework overlap with it. Figure 11b shows that the instant HRs estimated from the original signal (after bandpass filtering) is far from the ECG standard while the ones obtained using the proposed framework are close to it. More detailed evaluation and analysis is provided in Section 5.

## 5. Results and Discussion

The proposed algorithm is tested based on a dataset including 21 different types of motion from six measurement subjects. The performance of the algorithm is evaluated in terms of the error in average HR.

### 5.1. Evaluation and Performance Metrics

The reference HR is obtained from the simultaneously recorded ECG signal by time domain R peak detection. The average HR is calculated in 8 s sliding window with 7 s overlap and the instant HR is calculated on a beat to beat basis. There are two sets of performance metrics for average HR and instant HR.

The average absolute error (AE), defined in Equation (4), is used as the main metric for the error in average HR estimation. This equation takes the average error from N sliding windows. Within each window, the absolute value of the difference between the average HR estimated from the (pre)processed PPG (BPM_est_) and the average HR calculated by the reference ECG channel (BPM_ref_) is calculated. Like the average HR from the ECG signal, the HR derived from the PPG signal is calculated in 8 s sliding windows with 7 s overlap:

(4) $$AE=\frac{1}{N}\overset{N}{\underset{j=1}{\sum }}\left|BPM_{est}\left(j\right)-BPM_{ref}\left(j\right)\right|$$

### 5.2. Results and Discussions

The evaluation results together with discussions for periodic, random, and continuous non-periodic motion situations are given in Section 5.2.1, Section 5.2.2 and Section 5.2.3, respectively.

#### 5.2.1. Periodic Motion

**Figure 12:**
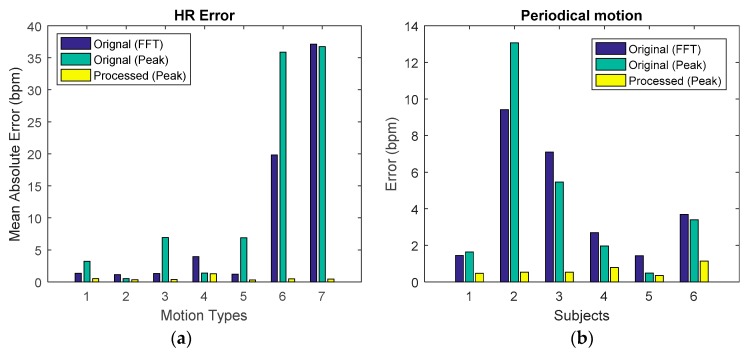
HR estimated in periodic motions after processing compared with preprocessing and ECG reference (**a**) HR estimation error for one subject in seven periodic motion situations. (**b**) HR estimation error for 6 subjects in periodic motion situations.

The proposed algorithm framework considerably reduced the AE of HR for periodic motion in all subjects, compared to AE of HR calculated using FFT or time domain peak detection after preprocessing (bandpass filtering), Figure 12b. In addition, Figure 12a shows the performance of the framework for the different types of periodic motion for one of the subjects. Again, the error in HR estimation was much smaller using the proposed framework compared to the error in HR estimation obtained using the FFT or time domain detection after band pass filtering. For all motion types, the proposed framework reduced the AE of HR to below 2 bpm, Figure 12a. These results suggest the applicability of the framework for reliable HR estimation for a wide range of periodic micromotions. 

#### 5.2.2. Random Motion

**Figure 13:**
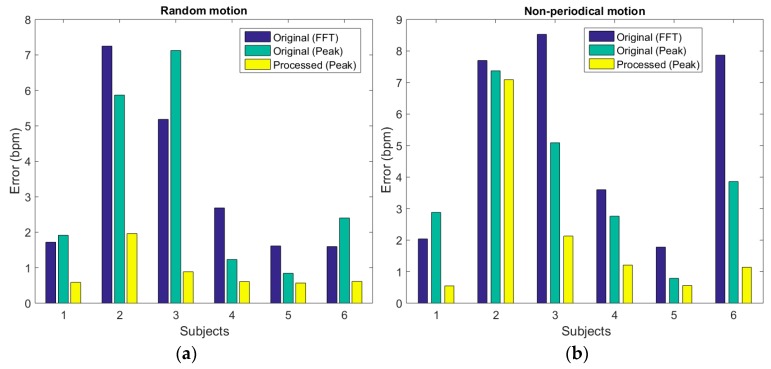
(**a**) HR estimation error for 6 subject in random motion situations. (**b**) HR estimation error for six subjects in continuous non-periodic motion situations.

For random motion, the average HR error is reduced to below 2 bpm for all six subjects after processing with the proposed framework, Figure 13a. This error is considerably smaller than the error in HR estimation using FFT or time domain detection after band pass filtering (Figure 13a). 

During random motion, the subjects could perform the specified motion non-periodically and with intervals. However, the MAR step using the CWT based subtraction performs best for periodic motion. The good performance of the proposed algorithm for random motion can be attributed to the frequency tracking step. This allows reliably detected HR frequency information from a segment with stationary conditions to be carried over to the next segment with motion. When the motion stops, the relative weight of the HR detection of the current segment will become larger again.

#### 5.2.3. Continuous Non-Periodic Motion

The evaluation results for continuous non-periodic motion are illustrated in Figure 13b. The AE of HR is reduced in all subjects, showing large reductions of the error to below or around 2 bpm for five subjects and a small reduction of the error for subject 2. This is most likely caused by differences in intensity of the continuous motion between subjects, with the continuous motion intensity of subject 2 being much higher than the intensity from the other subjects. 

As indicated, the CWT-based MAR method performs best for periodic motion. In case of continuous non-periodic motion, the MAR method can be less reliable, and the frequency tracking can start to fail, resulting in a reduced accuracy of the HR estimation. Importantly, time domain observation showed that the proposed framework can still perform well when the amplitude of the MA was similar to the amplitude of the HR component. The evaluation results on AE of HR are summarized in Table 1. The results obtained from preprocessed data (by FFT and time domain peak detection) are given in the 1st and 2nd row, while the results from the proposed framework are given in the 9th row. Table 1 also includes results obtained by applying other motion reduction methods and replacing certain step of the proposed algorithm framework, which will be discussed in Section 5.3.

### 5.3. Comparison with Other Methods and Internal Steps

The proposed algorithm is compared with three motion artifact reduction methods. Since, we are not aware of any papers using a different wavelength PPG channel as motion reference, the framework is compared to methods originally developed for a different motion reference and to ICA: (1) adaptive filtering [24]; (2) periodic motion tracking by Wijshoff [22]; and (3) independent component analysis (ICA) [25]. Methods (1) and (2) use the IR channel as the motion reference for MAR and method (3) treats the two channels similarly. In order to make a fair comparison, the motion detection step (discussed in Section 4.1) is also included in methods (1) and (2). The results in terms of average HR error are given in Table 1, where methods (1), (2) and (3) are expressed as CAdFMAR (AdFMAR), CTraMAR (TraMAR), and IdsSE, respectively. When the C is used as prefix for AdFMAR and TraMAR, the motion detection step proposed in the current paper is used in combination with the adaptive filter (AdFMAR) or the periodic motion tracking (TraMAR), respectively.

The proposed method is most effective in deriving the average HR from the PPG signal corrupted by micromotions (Table 1). In contrast, the focus of many papers on MAR for the PPG signal is on larger motions, like running. These types of movements, in contrast to micromotions, are well captured with an accelerometer. 

The much higher HR error obtained by adaptive filtering compared to the HR error obtained by our framework is caused by the lack of time domain correlation between the MA signals in green and the IR PPG as described in Section 3.2.1. Secondly, the fast changes of signal to motion ratio in the time domain also reduced the effectiveness of adaptive filtering. Finally, the adaptive filtering method was originally designed with use of the accelerometer as a motion reference [24]. The IR PPG signal contains both HR and motion information, in contrast to the accelerometer signal which contains motion information. When adaptive filtering with the IR PPG signal is applied to periodic motion, the HR information in the IR PPG signal will reduce the performance of the algorithm. 

The HR estimate obtained by periodic motion tracking method CTraMAR also gives a larger error than the error from the HR estimate from the proposed framework. The performance of this method is also negatively influenced by the HR component in the IR PPG signal. In addition to that, this method only works in periodic motion situations and not for random or continuous non-periodic movements. The results obtained by both the motion tracking and the adaptive filtering are clearly improved when combined with the motion detection (CAdfMAR, CTraMAR).

The results obtained by the ICA are less accurate than the results obtained by the proposed algorithm framework. The main reason for the lower accuracy could be that there are only two input signal channels (green and IR PPG). If more channels (more wavelengths) would be used, ICA could potentially be a good alternative MAR method. 

Finally, within the proposed framework, the SSA based signal reconstruction is compared to simply bandpass filtering the green PPG signal by the frequency mask, i.e., replacing the SSA step with a bandpass filter in the framework. The results are given in Table 1 as BpfSE. The results show larger error in the average HR. Compared to the SSA, the bandpass filtering with the frequency mask is highly dependent on the accuracy of the approximate HR estimation step, which causes a low fault tolerance. 

To summarize, the proposed method is most effective in removing artifacts induced by micromotions from PPG signals using IR PPG as a motion reference. The best results are observed for periodic micromotions. In addition, it also reduces the error in average HR estimation in random and non-periodic motion situations. The effectiveness of the proposed method is, mainly during non-periodic movement, dependent on the motion intensity (Figure 13b and Table 1). The error in the HR estimation with the proposed framework during continuous non-periodical motion was higher for subject 2, 3, 6 compared to random motion situations as shown in Figure 13. This results in a higher average error and standard deviation for this motion type compared to the other motion types in Table 1. The results discussed in this section show that the proposed framework is most effective, compared to existing solutions, in removing MA from the green PPG when the IR PPG is used as motion reference.

## 6. Conclusions and Recommendations

In this study, a framework for MAR from green PPG signal using IR PPG signal as a reference was proposed and tested in a dataset with focus on daily living micromotions. The framework was built based on the features of the motion and PPG components in the green and IR signals. The proposed algorithm framework reduces the average error in HR from 4.3 bpm, 3.0 bpm and 3.8 bpm to 0.6, 1.0 and 2.1 bpm in periodic, random, and continuous non-periodic motion situations respectively. It is an effective algorithm for MAR using green and IR PPG signals based on our dataset, when compared to several methods.

This work is the first attempt to remove MA in wrist PPG signals, by using another PPG channel with a different wavelength as the motion reference. The wavelengths are chosen based on signal properties in a limited dataset. A comprehensive study with more wavelengths and motion types can provide a better understanding of the motion signal recorded by different wavelengths. A more effective motion reference may be obtained, e.g., another wavelength or a combination of several wavelengths. Finally, for further validation of the framework a dataset with a larger number of measurement subjects and additional motion types should be used. Nevertheless, the evaluation and comparison results indicate that the proposed method is effective in reducing the error in HR estimation from PPG signals with periodic, random and non-periodic motion artifacts during daily life.

## Figures and Tables

**Table 1:** Error in estimated HR (mean ± standard deviation in bpm) after processing with different methods.

Method type		Motion Detection	Method Name	Periodic	Random	Cont. Non-Periodic
Preprocessing		No	FFT	4.3 ± 3.3	3.3 ± 2.4	5.3 ± 3.1
			Peak detection	4.3 ± 4.6	3.0 ± 2.3	3.8 ± 2.3
Existing motion artifacts removal methods	Motion reference	No	AdfMAR	4.5 ± 2.6	3.7 ± 1.7	3.2 ± 1.4
			TraMAR	3.8 ± 3.4	-	-
		Yes	CAdfMAR	2.8 ± 2.5	2.8 ± 2.2	3.0 ± 1.5
			CTraMAR	3.4 ± 3.5	-	-
	Multichannel PPG	N.A.	IdsSE	4.7 ± 1.9	4.1 ± 0.8	4.6 ± 2.1
Proposed framework	With bandpass filter	Yes	BpfSE	0.9 ± 0.3	1.4 ± 1.0	2.8 ± 3.0
	Original	Yes	Proposed	0.6 ± 0.3	0.9 ± 0.6	2.1 ± 2.5

FFT: peak detection using FFT after preprocessing, Peak detection: time-domain peak detection after preprocessing, AdFMAR: Adaptive Filtering based MAR [24], CAdFMAR: Adaptive Filtering based MAR [24] with motion detection, TraMAR: Track motion frequency-based MAR [22], CTraMAR: Track motion frequency-based MAR [22] with motion detection. IdsSE: Direct signal decomposition with independent component analysis (ICA), BpfSE: Replace the SSA decomposition and reconstruction by bandpass filtering using the frequency mask, Proposed the proposed framework.
